# Tetrodotoxin-Bupivacaine-Epinephrine Combinations for Prolonged Local Anesthesia

**DOI:** 10.3390/md9122717

**Published:** 2011-12-15

**Authors:** Charles B. Berde, Umeshkumar Athiraman, Barak Yahalom, David Zurakowski, Gabriel Corfas, Christina Bognet

**Affiliations:** 1 Department of Anesthesiology, Perioperative and Pain Medicine, Children’s Hospital Boston, Harvard Medical School, 300 Longwood Avenue, Boston, MA 02115, USA; Email: ukathiraman@gmail.com (U.A.); barak.yahalom@gmail.com (B.Y.); david.zurakowski@childrens.harvard.edu (D.Z.); cbognet@alum.mit.edu (C.B.); 2 Departments of Otolaryngology and Neuroscience, Children’s Hospital Boston, Harvard Medical School, 300 Longwood Avenue, Boston, MA 02115, USA; Email: gabriel.corfas@childrens.harvard.edu

**Keywords:** tetrodotoxin, bupivacaine, epinephrine, local anesthesia, sciatic blockade

## Abstract

Currently available local anesthetics have analgesic durations in humans generally less than 12 hours. Prolonged-duration local anesthetics will be useful for postoperative analgesia. Previous studies showed that in rats, combinations of tetrodotoxin (TTX) with bupivacaine had supra-additive effects on sciatic block durations. In those studies, epinephrine combined with TTX prolonged blocks more than 10-fold, while reducing systemic toxicity. TTX, formulated as Tectin, is in phase III clinical trials as an injectable systemic analgesic for chronic cancer pain. Here, we examine dose-duration relationships and sciatic nerve histology following local nerve blocks with combinations of Tectin with bupivacaine 0.25% (2.5 mg/mL) solutions, with or without epinephrine 5 µg/mL (1:200,000) in rats. Percutaneous sciatic blockade was performed in Sprague-Dawley rats, and intensity and duration of sensory blockade was tested blindly with different Tectin-bupivacaine-epinephrine combinations. Between-group comparisons were analyzed using ANOVA and *post-hoc* Sidak tests. Nerves were examined blindly for signs of injury. Blocks containing bupivacaine 0.25% with Tectin 10 µM and epinephrine 5 µg/mL were prolonged by roughly 3-fold compared to blocks with bupivacaine 0.25% plain (*P* < 0.001) or bupivacaine 0.25% with epinephrine 5 µg/mL (*P* < 0.001). Nerve histology was benign for all groups. Combinations of Tectin in bupivacaine 0.25% with epinephrine 5 µg/mL appear promising for prolonged duration of local anesthesia.

## 1. Introduction

Currently available local anesthetics typically do not provide median durations of analgesia lasting more than 12 hours following wound infiltration, peripheral nerve blockade, or plexus blockade [1,2,3]. Duration of analgesia can be prolonged by use of indwelling catheters, but these have some potential for dislodgment and infection, and are inconvenient in some locations in the body. Elastomeric pumps are used for home infusions, however inconsistent delivery has been reported [4]. Since pain is often intense for several days following surgery, it would be quite useful to have local anesthetics that could more reliably provide analgesia for 18–48 hours following a single injection. 

The site 1 sodium channel toxins, including tetrodotoxin (TTX), block impulse conduction in isolated nerves with high potency. In overdosage, as occurs with poisoning from sushi derived from puffer fish, they can produce respiratory muscle weakness and hypotension [5,6]. Unlike existing local anesthetics, these toxins do not cause direct myocardial depression, and they cross the blood brain barrier very poorly, reducing their risk of seizures or central nervous system depression. 

Tetrodotoxin (TTX) is currently undergoing Phase III clinical trials in Canada as a systemic analgesic for inadequately controlled pain due to advanced cancer, especially where the pain has neuropathic features [7,8]. The TTX formulation in these trials, known as Tectin, contains TTX at a concentration of 15 µg/mL (47 µM), in 2 mL ampoules for subcutaneous injection. Human subjects have tolerated doses of up to 90 µg daily, with mostly mild side-effects observed at doses below 60 µg. 

We have previously shown that prolonged duration percutaneous sciatic nerve blockade could be achieved in rats using combinations of site 1 sodium channel toxins with either bupivacaine or epinephrine [9]. Remarkably, epinephrine 10 µg/mL prolonged the duration of TTX blockades by more than 10-fold [9]. Addition of either bupivacaine or epinephrine increases the LD_50_ of TTX in rats, *i.e.*, reduces systemic toxicity from TTX, and also increases the potency of TTX for producing sensory blockade, thereby substantially improving the therapeutic index of TTX. Thus, combination formulations seem desirable from the standpoint of both efficacy and safety.

The toxicity of TTX relates to two factors: paralysis of skeletal muscle, including the diaphragm and intercostal muscles, leading to respiratory failure, and reduced blood pressure, predominantly due to vasodilatation. Early signs of this effect might include weakness, tingling of the lips, dizziness, *etc.* The toxicity of TTX is much lower by oral or inhaled routes compared to by injection. Previous measurements of LD_50_ in mice, rats, dogs and rabbits following intramuscular injection have ranged 10–20 μg/kg. In anesthetized dogs, TTX up to 4 μg/kg intramuscularly did not have any effect on the blood pressure, ECG, or heart and respiratory rates. 

Unlike bupivacaine and other currently used local anesthetics, TTX does not cause direct myocardial depression or arrhythmias because it does not bind significantly to Nav 1.5 sodium channels, the predominant subtype found in the heart. In addition, TTX crosses the blood-brain barrier poorly, and does not generate seizures. Necropsy of the experimental animals did not show any abnormalities in organs or staining of the sciatic nerve. Based on these results, we believe that TTX can be a systemically safe drug over a particular dose range.

In different clinical contexts, local anesthetics may be used in relatively large volumes (e.g., up to 90 mL for abdominal wound infiltration) or relatively small volumes (e.g., as little as 12 mL for ultrasound-guided interscalene block). In our view, a convenient, flexible, and safe approach to clinical development would be to supply clinicians with a standard package containing two vials, a 2 mL ampoule of TTX as Tectin along with a larger 30 mL vial of bupivacaine 2.5 mg/mL (0.25%) with epinephrine 5 µg/mL (1:200,000). This would permit the clinician to mix the two vials immediately prior to use in different ratios for different clinical indications. 

Prior to planned human studies, in the current study, we used the rat percutaneous sciatic nerve block model to evaluate duration of sensory blockade with combinations of Tectin with bupivacaine 0.25%, with or without epinephrine 1:200,000. In considering clinical development of a mixture with two active anesthetics and one additive, it is important to provide preclinical data that confirms the contribution of each drug in the combination from the standpoint of both efficacy and of safety. 

For comparison purposes, block durations in humans are generally 2–4 fold longer than the durations seen with comparable weight-scaled volumes and concentrations in rats [1,2,10]. 

Tectin has been formulated as a sterile buffered aqueous solution containing 15 µg/mL TTX for parenteral administration. For comparison purposes, parallel neurobehavioral experiments were performed with highly purified TTX derived from another commercial supplier (Sigma-Aldrich Chemicals, St. Louis, MO), as was used in previous publications. Previous studies have found TTX and other site 1 toxins to be histologically benign on nerves and muscles [9,11]. In considering planned human study of Tectin for perineural injection, rat sciatic nerves were examined histologically for signs of injury to nerve fibers. 

We hypothesized that:

(1) TTX alone is suboptimal as a local anesthetic at doses devoid of systemic analgesia, sedation or motor impairment: combination formulations are required to achieve optimal safe and well-tolerated prolonged-duration local anesthesia;
(2) Addition of TTX to bupivacaine 0.25%, with or without epinephrine, results in longer-duration blocks compared to corresponding bupivacaine-containing solutions without TTX;
(3) Addition of epinephrine to bupivacaine-TTX mixtures results in longer duration blocks than corresponding bupivacaine-TTX mixtures without epinephrine;
(4) Block-prolongation from TTX as Tectin is similar to that observed with TTX obtained from Sigma; and
(5) Histological effects on rat sciatic nerves of Tectin, alone and in combination with bupivacaine and epinephrine, are benign.

## 2. Results and Discussion

### 2.1. Results

#### 2.1.1. Neurobehavioral Studies (Summarized in Figure 1)

**Figure 1:**
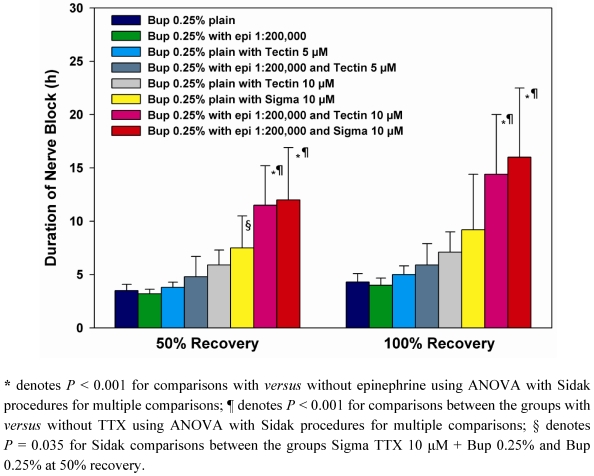
Duration of thermal nocifensive sensory block. Block durations are depicted for time to 50% and 100% recovery, as defined in the Experimental section. Data are presented as mean + SD. As detailed in the Results, addition of tetrodotoxin (TTX) (derived from WEX or Sigma formulations) to combinations of bupivacaine 0.25% with epinephrine prolongs blocks compared to bupivacaine 0.25% without TTX. Epinephrine prolongs blocks from combinations of bupivacaine with TTX (from either source) compared to solutions containing bupivacaine and TTX without epinephrine.

#### 2.1.2. Rat Sciatic Nerve Blockade *in Vivo* with TTX Alone

As noted in the Experimental section, pilot experiments (*n* = 3) reconfirmed that blocks with TTX 20 µM produced short-duration sedation and prolongation of contralateral thermal withdrawal latencies, but did not achieve complete thermal nocifensive blockade, *i.e.*, ipsilateral withdrawal latencies were always less than 12 seconds, giving blocks of zero duration. Injections with TTX 10 µM alone (*n* = 5) did not produce sedation or prolongation of contralateral latencies, also producing blocks of zero duration. 

#### 2.1.3. Effects of TTX on Duration of Rat Sciatic Nerve Blockade *in Vivo*

GLM-ANOVA showed an overall effect of TTX 10 µM in prolonging block durations (for 50% recovery, *F* = 7.05, *P* < 0.001; for 100% recovery, *F* = 6.58, *P* < 0.001). Formulations without TTX, *i.e.*, those containing bupivacaine 0.25%, with or without epinephrine, all gave mean durations for 50% or 100% recovery of 4 hours or less (Figure 1). In formulations containing epinephrine, addition of TTX 10 µM resulted in 3-fold prolongation in block durations compared to corresponding formulations without TTX 10 µM, and these prolongations were statistically significant by Sidak *post-hoc* testing at the *P* < 0.001 level (Figure 1). Addition of TTX 10 µM to bupivacaine 0.25% without epinephrine did not result in statistically significant prolongation of block durations compared to bupivacaine 0.25% alone (Figure 1). 

#### 2.1.4. Effects of Epinephrine on Duration of Rat Sciatic Nerve Blockade *in Vivo*

GLM-ANOVA showed an overall effect of epinephrine in prolonging block durations (for 50% recovery, *F* = 18.75, *P* < 0.001; for 100% recovery, *F* = 15.18, *P* < 0.001). As shown in Figure 1, in the absence of TTX, epinephrine had no block prolonging effect on bupivacaine 0.25%. In the presence of bupivacaine and TTX, epinephrine resulted in 1.6–1.9-fold prolongation of block durations for 50% recovery and 1.7–2-fold prolongation of block durations for 100% recovery compared to bupivacaine and TTX without epinephrine (*P* < 0.001 by Sidak’s *post-hoc* tests). 

#### 2.1.5. Effect of Commercial Source of TTX on Block Durations

GLM-ANOVA showed no significant effect of commercial source of TTX on block durations. In all combinations, TTX as Tectin (formulated in an acetic acid-sodium acetate buffer) behaved indistinguishably from Sigma TTX (Figure 1). 

#### 2.1.6. Additional Behavioral Observations

No animals were observed to have swelling at the injection sites, or signs of distress, hyperalgesia, or self-mutilation. No animals had any evident residual weakness or impairment of gait when re-assessed at 24 hours following the injections. 

#### 2.1.7. Histology

Histologic injury scores are summarized in Table 1. There were no significant overall effects of treatment condition on the scores for myelinated axons or perineural tissues. Overall, the distribution of scores appeared benign, and the opinion of the examiner was that the occasional appearance of mild injury in perineural tissues (occasional scores of 2 or 3) could have been related to needle trauma or fixation artifact. Additionally, there were no visible signs of inflammation noted. 

**Table 1:** Comparison of Histologic Injury Scores Between Treatments. Data represent median values with interquartile ranges shown in parentheses. Nonparametric Kruskal-Wallis test indicated no significant overall effect of treatment on histologic injury scores for myelinated axons or for perineural tissues viewed at 10X or 60X.

Treatment (*n* = 6 for Each Condition)	Myelinated Axons 10X	Myelinated Axons 60X	Perineural 10X	Perineural 60X
Bup + Epi	1 (1-1)	1 (1-2)	1 (1-2)	1 (1-2)
Bup+Epi+Tectin 10 μM	1 (1-2)	1 (1-2)	1 (1-2)	2 (2-2)
Tectin-free Placebo-Vehicle	1 (1-2)	1 (1-2)	1 (1-1)	1 (1-1)
Tectin 10 μM	1 (1-1)	1 (1-2)	1 (1-2)	1.5 (1-2)
Contralateral	1 (1-1)	1 (1-2)	1 (1-1)	1 (1-1)

Bup = bupivacaine; Epi = epinephrine.

**Figure 2:**
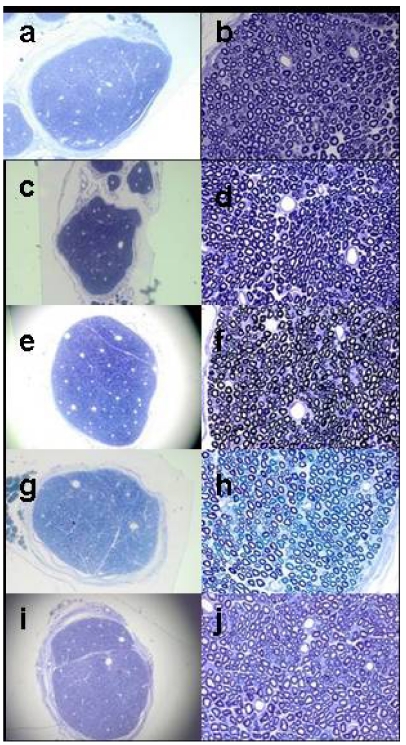
Histology of Sciatic Nerves. Representative photomicrographs of sciatic nerve sections harvested one week after injection. Slides were coded and blindly examined for injury scores at 10X (left column, panels **a**,**c**,**e**,**g**,**i**) and 60X (right column, panels **b**,**d**,**f**,**h**,**j**) magnifications as analyzed in Table 1. Treatment conditions shown were bupivacaine 0.25% with epinephrine 1:200,000 (**a**,**b**); bupivacaine 0.25% with epinephrine 1:200,000 and Tectin 10 µM (**c**,**d**); Tectin-free Vehicle mixed 50-50 with preservative-free normal saline (**e**,**f**); Tectin 10 µM diluted with preservative-free normal saline (**g**,**h**); and Contralateral (right) uninjected control sciatic nerves (**i**,**j**).

### 2.2. Discussion

Combination local anesthetic formulations containing bupivacaine, TTX, and epinephrine appear promising for prolonged-duration of local anesthesia. Neurobehavioral experiments support the inclusion of all three drugs in the mixture as an approach to prolong block durations while minimizing the systemic toxicity of either TTX or bupivacaine. This three-drug “high bupivacaine-low TTX-concentration” combination is likely to be especially important for clinical situations such as wound infiltration for a large open abdominal incision. For this application, very large injection volumes (e.g., 60–90 mL in adults) are required to provide blockade of afferents from multiple layers of the wound, and therefore TTX concentrations must be low in order to keep the total systemic dose in a safe range. The three drug combination is also likely to improve the reliability of blockade compared to formulations using TTX-epinephrine without bupivacaine.

In this study, Tectin appeared indistinguishable from TTX supplied by Sigma in its neurobehavioral effects, and Tectin and its placebo-vehicle appeared benign in its histologic effects on rat sciatic nerves. 

Peripheral nerve blockade and wound infiltration can provide useful contributions to multimodal approaches to postoperative recovery [12]. Currently, prolonged peripheral nerve blockade and prolonged wound-analgesia can be supplied by indwelling catheters and infusion pumps. Catheters can become dislodged or can unintentionally preferentially block one trunk, cord, or division of a plexus. While portable elastomeric infusion pumps are convenient, and can facilitate local anesthetic delivery at home, they incur some expense, and some studies indicate that delivery failures are not rare [4]. In our view, an approach that could reliably prolong the duration of peripheral nerve blockade or infiltration analgesia by 3–4 fold would be enormously important for postoperative care. Since both the desirable effect (analgesia) and other effects (e.g., motor blockade) are greatly prolonged, in future clinical development, it will be important to restrict injections to body locations where motor effects are clinically unimportant.

## 3. Experimental Section

### 3.1. Reagents

All drug combinations used for sciatic nerve injection were mixed immediately prior to use. All samples were coded so that investigators performing sciatic blockade and behavioral testing were blinded to the contents of the injectable solutions. 

Tectin ampoules (clinical grade) were supplied by WEX Pharmaceuticals, Vancouver, B.C., Canada. These ampoules contain 30 µg TTX in 2 mL of an aqueous vehicle containing acetic acid-sodium acetate buffer, pH 3.5–4.5. Parallel experiments used crystalline TTX obtained from Sigma Chemical Co. (St. Louis, MO, USA). TTX-containing solutions were stored in a monitored temperature-controlled cold room at 4 °C prior to use. Placebo ampoules containing the acetic acid-sodium acetate buffer, pH 3.5–4.5 without any active Tectin were also supplied by WEX Pharmaceuticals, Vancouver, B.C., Canada. After an ampoule was opened, it was transferred to a screw-capped tube and stored at 4 °C for sub-aliquoting in subsequent experiments for no more than 1 week. Commercial solutions of bupivacaine 0.25% with (Hospira, Lake Forest, IL, USA) or without (Astra-Zeneca, Wilmington, DE, USA) epinephrine 1:200,000 (5 µg/mL) were opened fresh prior to aliquoting for each day’s experiments. Commercially-determined expiration dates were checked to verify that no outdated solutions were used. Preservative-free normal saline solution was used fresh for each day’s experiments.

### 3.2. Animal Care

Animals were cared for in compliance with protocols approved by the Children’s Hospital Animal Care and Use Committee. Young adult male Sprague-Dawley rats weighing 200–300 g were obtained from Charles River Laboratories (Wilmington, MA) and were housed in groups and kept in a 7 a.m. to 7 p.m. light-dark cycle. Rats were handled repeatedly by the investigators to diminish effects resulting from stress-induced analgesia. 

### 3.3. Sciatic Blockade Technique

Procedures for sciatic blockade and neurobehavioral testing were as described previously [9,13]. General anesthesia was induced in a chamber with isoflurane 5% in 100% oxygen for one to two minutes. Animals were then removed from the chamber for percutaneous sciatic blocks. The duration of loss of righting reflex was no more than 3 minutes. Previous studies indicated that brief exposure to general anesthesia reduced aversive behaviors with repeated sensory testing, made injection more precise, and had no effect on sensory thresholds measured 5 minutes later. Percutaneous sciatic nerve blockade is performed by injecting a 0.3 mL volume from a 1 mL N-100 insulin-type syringe and 30 gauge 1.3 cm needle. In an injection volume of 0.3 mL, 10 micromolar TTX gives a dose of 0.96 microgram. The left leg was always used for blocks, the right leg served as a control. Blocks are performed by right handed investigators using left-thumb-palpation of the left greater trochanter, by introducing the needle posteromedially to the greater trochanter pointed in an anteromedial direction. Upon contacting the ischium, the needle was withdrawn 1 mm and the drug was injected. Previous experience indicates that investigators can achieve >95% success rates even with 0.1 mL injections and >98% success rates with 0.3 mL injections. Based on this experience, for data analysis, we regarded blocks of apparent zero duration (operationally defined below) as pharmacologically ineffective solutions, rather than as technical failures. 

Syringes are coded and blinded by an investigator uninvolved in the injections or in the neurobehavioral testing. 

### 3.4. Neurobehavioral Assessment of Nerve Blockade: Modified Hotplate Test to Test Thermal Sensory (Nocifensive) Block

Blockade of thermal nociception (TN) was assessed using a modified hot plate test, developed by Thalhammer *et al.* [9,13]. Briefly, rats were held upright with the investigator’s hands around the thorax and abdomen, and lowered so that the lateral aspect of a single hindpaw (alternately right or left) was touching a hot plate at 56 °C (model 39D hot plate analgesia meter; IITC Inc., Woodland Hills, CA, USA), and the time (thermal latency) until the animal withdrew its paw was measured with a stopwatch. If the rat did not lift the paw after 12 seconds, the paw was removed by the experimenter to avoid injury to the animal or the development of hyperalgesia. 

The contralateral (right) hindpaw is also tested as a control for systemic analgesia, systemic sedation, or systemic weakness due to TTX effect. Pilot experiments confirmed that mixtures containing TTX as Tectin 20 µM produced brief (<30 minutes) prolongation of contralateral thermal withdrawal latencies, consistent with previous studies. For this reason, subsequent experiments restricted the concentration of TTX to no more than 10 µM. At this concentration, no prolongation of contralateral thermal withdrawal latencies was observed. 

### 3.5. Histological Examination

Five treatment conditions were used for histology, *n* = 6 for each group.

Group 1 Bupivacaine 0.25% with epinephrine 1:200,000;
Group 2 Bupivacaine 0.25% with epinephrine 1:200,000 and Tectin 10 µM;
Group 3 Tectin-free (placebo) Vehicle mixed 50-50 with preservative-free normal saline;
Group 4 Tectin 10 µM diluted with preservative-free normal saline;
Group 5 Contralateral (right) uninjected control sciatic nerves.

Rats received sciatic injections with the treatment groups as mentioned above, and they were allowed to recover in their cages. All rats in Groups 1 and 2 received successful blocks, as determined by signs of sensory blockade per above. Groups 3 and 4 did not show signs of sensory blockade, as expected. Seven days following injections, they were terminally anesthetized with pentobarbital 100 mg/kg, and underwent cardiac puncture for perfusion with cold phosphate buffered saline 30 mL, followed by 100 mL of cold fixative, containing 1.25% PFA, 2.5% GA, 0.03% Picric Acid in 0.1 M Cacodylate buffer. 

Sciatic nerves were dissected, preserved in the fixative for 48 hours, washed 2X in 0.1 M cacodylate buffer, and post fixed with 1% OsO_4_ in 0.1 M cacodylate buffer for 48 hours. After fixation, sciatic nerves were washed 2X for 1 hour in 0.1 M cacodylate buffer, dehydrated in graded ethanol solutions: 50, 75, 95, 100% (2X), and suspended in Propylene Oxide for 1 hour 2X. Before embedding the nerve fragments in plastic resin, they were suspended in solution of (Propylene Oxide:araldite-ddsa) 1:1: overnight ,and serial sections of 1 µm thickness were cut with an ultra-microtome (Reichert Ultracuts, Leica AG 702501, Austria). Slides were coded and blindly examined for axonal injury or demyelination at 10X and 60X magnification with the light microscope (Nikon Eclipse E800) by a senior neuroscientist with expertise in evaluation of peripheral nerves (Gabriel Corfas) who remained blinded to treatment conditions. Histological samples of sciatic nerves based on representative photomicrographs one week after harvesting according to treatment are shown in Figure 2. 

Ratings for injury to nerve fibers were as described in a manner similar to Estebe and Myers [14], with the following 0–5 point ordinal Likert scale: 0 = no pathology in any portion of the field, 1 = very few myelinated axons with any mild abnormality, 2 = slightly more myelinated axons with any abnormality than 1, but <10% abnormal, 3 = 10–20% abnormal myelinated axons, 4 = signs of moderate axonal degeneration, and 5 = clear signs of axonal degeneration. An analogous 0–5 scale was used for scoring perineural tissues. 

### 3.6. Statistics

Data are reported in Table 1 as medians and interquartile ranges. Data in Figure 1 are depicted as mean values, and error bars denote standard deviations. With the modified hotplate test using a 56 degree hotplate, unblocked animals at baseline typically remove their hindpaws in 2 or 3 seconds. With dense sensory blockade, hindpaws are removed from the hotplate after 12 seconds exposure to avoid thermal injury or hyperalgesia; 12 seconds latencies are taken to show dense thermal nocifensive sensory blockade. Time to 50% recovery was defined as time to return to 7 seconds withdrawal latencies and time to complete recovery as the time to return to that individual animal’s pre-block baseline latency. 

Analysis of variance (ANOVA) using a generalized linear models (GLM) approach was used to compare duration of nerve block between the different treatments with a significant overall *F*-test followed by the powerful Sidak-Bonferroni procedure to adjust the *P*-values for multiple comparisons. Mean block duration was determined with a 95% confidence interval (CI) as a measure of precision around the observed duration for each treatment. *P*-values are 2-tailed with the Sidak adjustment applied to control the Type-I familywise error rate for multiple tests of hypotheses by conservatively adjusting the criterion for significance based on the number of planned comparisons [15].

GLM-ANOVA was used to examine for: (1) differences between formulations containing TTX as Tectin supplied by WEX compared to TTX prepared from Sigma (2) An effect of TTX on block durations in comparison to the same formulations without TTX, and (3) an overall effect of epinephrine on block durations. Wald chi-square tests were used to assess significance and confidence intervals. Sidak corrections were used for *post-hoc* between-group comparisons in Figure 1. 

For histologic injury scores, which are rank-ordered, the nonparametric Kruskal-Wallis test was used to examine overall group differences, as shown in Table 1. If an overall effect of group on scores had been found, *post-hoc* comparisons would have used corrected Mann-Whitney *U*-tests for between group comparisons. All statistical analysis was performed using the SPSS software package [16]. 

## 4. Conclusions

Three-way combinations of TTX, bupivacaine and epinephrine produce significant prolonged sciatic nerve blockade in rats, compared to bupivacaine plain or bupivacaine + epinephrine. Tetrodotoxin, formulated as Tectin, is currently in Phase III trials in Canada as an analgesic for chronic cancer pain. Using rat sciatic nerve blockade, the experiments reported here support the view that clinically-relevant combinations of Tectin with commercially-available bupivacaine 0.25% and epinephrine 5 µg/mL warrant further study for prolonged-duration local anesthesia. Our belief is that a single-injection approach to prolonged local anesthesia will provide good pain relief as a component of multi-modal analgesic regimens, will thereby reduce postoperative opioid requirements, and may improve the course of postoperative recovery and acute rehabilitation.
